# Healthy Adults Display Long-Term Trait-Like Neurobehavioral Resilience and Vulnerability to Sleep Loss

**DOI:** 10.1038/s41598-017-14006-7

**Published:** 2017-11-02

**Authors:** Laura E. Dennis, Rachael J. Wohl, Lauren A. Selame, Namni Goel

**Affiliations:** Division of Sleep and Chronobiology, Department of Psychiatry, University of Pennsylvania Perelman School of Medicine, Philadelphia, PA 19104 USA

## Abstract

Sleep loss produces well-characterized cognitive deficits, although there are large individual differences, with marked vulnerability or resilience among individuals. Such differences are stable with repeated exposures to acute total sleep deprivation (TSD) within a short-time interval (weeks). Whether such stability occurs with chronic sleep restriction (SR) and whether it endures across months to years in TSD, indicating a true trait, remains unknown. In 23 healthy adults, neurobehavioral vulnerability to TSD exposures, separated by 27–2,091 days (mean: 444 days; median: 210 days), showed trait-like stability in performance and subjective measures (82–95% across measures). Similarly, in 24 healthy adults, neurobehavioral vulnerability to SR exposures, separated by 78–3,058 days (mean: 935 days; median: 741 days), also showed stability (72–92% across measures). Cognitive performance outcomes and subjective ratings showed consistency across objective measures, and consistency across subjective measures, but not between objective and subjective domains. We demonstrate for the first time the stability of phenotypic neurobehavioral responses in the same individuals to SR and to TSD over long-time intervals. Across multiple measures, prior sleep loss responses are strong predictors of individual responses to subsequent sleep loss exposures chronically or intermittently, across months and years, thus validating the need for biomarkers and predictors.

## Introduction

The differences among healthy people in neurobehavioral decrements in response to sleep loss are large (i.e., order of magnitude) and stable over time^1–6^. Among healthy adults, approximately a third show profound performance deficits with even moderate sleep loss; a third show moderate deficits, and a third show few or no performance deficits, even when sleep loss is severe^7–9^. Thus, short-term trait-like (phenotypic) susceptibility among individuals accounts for 50–95% of the variance (depending on the measure) in the severity of neurobehavioral decrements due to sleep loss^7–9^. Re-exposure to acute total sleep deprivation (TSD) after one to six weeks reveals differential neurobehavioral vulnerability in various measures sensitive to sleep loss^1,3,10,11^. By contrast, no study has examined the stability of repeated exposure in the same subjects to chronic sleep restriction (SR), a commonly experienced type of sleep loss. In addition, longer time intervals, on the order of years, are needed to establish whether these responses to TSD and to SR are truly phenotypic and stable across time, when other factors change in individuals.

Stable and trait-like interindividual differences are observed in physiological responses to TSD, particularly in polysomnographic sleep and slow-wave energy responses to sleep loss across 2–3 days^12–14^, as well as in heart rate, heart rate variability, percentage of eyelid closure (PERCLOS), blink rate, and EEG alpha power across 2.5–15 months^15^. Furthermore, energy balance responses to sleep loss are phenotypic and stable across long time intervals of up to 2,132 days^16^ and across different types of sleep loss with 4 days between exposures^17^ (i.e., acute TSD and chronic SR).

Some studies have found task-dependent variability in response to acute TSD, with differential susceptibility across cognitive domains as measured by different neurobehavioral tests^10,18–21^. Further research is needed to understand the relationships between performance outcomes on different cognitive tasks and between subjective and objective measures of sleepiness and fatigue with respect to vulnerability and resistance to sleep loss across long-duration time intervals.

We sought to address three gaps in prior research: 1. We determined whether trait-like neurobehavioral response deficits are maintained over longer time intervals (months to years) between acute TSD exposures; 2. We determined for the first time whether chronic SR responses  show stability between repeated exposures; 3. We determined the relationships among various cognitive performance measures and subjective measures of sleepiness and fatigue across long-duration intervals and different types of sleep loss. We hypothesized an individual’s vulnerability or resistance to TSD and to chronic SR would remain highly stable between repeated exposures separated by long-duration time intervals. We also hypothesized objective performance measures would be related and subjective measures would be related, but that measures would not be related across cognitive and subjective domains for different types of sleep loss.

## Methods

### Subjects

A total of 23 healthy adults (12 women and 11 men, 21–50 years old, mean ± SD age at first study: 32.9 ± 8.1 years) participated in two acute TSD experiments separated by 27–2,091 days (Table 1; mean: 444 days; median: 210 days). A total of 24 healthy adults (10 women and 14 men, 21–50 years old, mean ± SD age at first study: 32.3 ± 8.4 years) participated in two chronic SR experiments separated by 78–3,058 days (Table 2; mean: 935 days; median: 741 days).

**Table 1:** Study Sample Characteristics for the TSD Protocols.

TSD Subjects	Age		Gender	Days Between Studies
	Exposure 1	Exposure 2		
A	35	35	F	27
B	40	40	M	41
C	32	32	M	71
D	22	22	M	77
E	33	33	F	82
F	32	32	F	110
G	28	28	M	117
H	35	35	F	129
I	42	42	F	153
J	21	22	F	156
K	40	40	M	185
L	25	26	F	210
M	27	28	M	213
N	37	38	M	224
O	41	42	F	229
P	48	49	F	235
Q	47	48	M	351
R	22	23	F	495
S	28	30	M	872
T	26	29	M	1104
U	23	27	F	1352
V	41	45	F	1696
W	32	38	M	2091

**Table 2:** Study Sample Characteristics for the SR Protocols.

SR Subjects	Age		Gender	Days Between Studies
	Exposure 1	Exposure 2		
A	25	25	F	78
B	22	22	M	118
C	25	26	M	194
D	42	42	F	213
E	32	32	M	224
F	32	33	F	249
G	46	46	M	261
H	25	26	F	263
I	27	28	M	282
J	30	31	F	317
K	37	38	M	353
L	22	23	F	590
M	47	50	M	891
N	28	31	M	904
O	38	40	M	923
P	26	29	M	1152
Q	26	30	M	1643
R	22	27	F	1666
S	23	28	F	1690
T	36	41	M	1744
U	40	45	F	1757
V	45	50	M	1758
W	43	49	F	2111
X	35	43	M	3058

In order to be eligible for study participation, subjects met the following inclusionary criteria: age range from 21–50 years; physically and psychologically healthy, as assessed by physical examination and history; no clinically significant abnormalities in blood chemistry; drug-free urine samples; good habitual sleep, between 6.5–8.5h daily duration with regular bedtimes and wake up times between 0600h–0900h (verified by sleep logs and wrist actigraphy for at least one week before study entry); absence of extreme morningness/eveningness, as assessed by questionnaire^22^; absence of sleep or circadian disorders, as assessed by questionnaire^23^ and polysomnography; no history of psychiatric illness and no previous adverse neuropsychiatric reaction to sleep deprivation; no history of alcohol or drug abuse; and no current use of medical or drug treatments (excluding oral contraceptives). The protocols were approved by the Institutional Review Board of the University of Pennsylvania, and all protocol methods were carried out in accordance with approved guidelines and regulations. Subjects provided written informed consent, which was in accordance with the Declaration of Helsinki. Subjects received compensation for participating in the protocols.

### Procedures

The experiments took place in a controlled environment in the Sleep and Chronobiology Laboratory at the Hospital of the University of Pennsylvania. During wakefulness, subjects were ambulatory and were permitted to perform sedentary activities such as watching television, reading, and playing video or board games between cognitive test bouts (completed while seated at a computer); however, they were not allowed to exercise. Ambient light was fixed at <50 lux during scheduled wakefulness, and <1 lux (darkness) during scheduled sleep, and did not differ across laboratory experiments. Ambient temperature was maintained at 22 ± 1 °C. Subjects were continuously monitored by trained staff. Subjects received three standardized meals per day, plus an optional healthy evening snack. Intake of caffeine, turkey, bananas, alcohol and tobacco was prohibited.

In each TSD experiment, during the laboratory phase, subjects received 1–2 baseline (9h–12h time-in-bed [TIB]) nights followed by 36h awake (acute TSD). In each SR experiment, during the laboratory phase, subjects received 2–3 baseline (8h–12h TIB) nights followed by 5 consecutive SR nights (4h TIB).

#### Neurobehavioral Measures

A computerized neurobehavioral test battery was administered every 2h during wakefulness and contained the following tasks: the 10-minute Psychomotor Vigilance Test (PVT)^24,25^, the Digit Symbol Substitution Task (DSST)^26^, the Forward and Backward Digit Span Task (DS)^26^, the Karolinska Sleepiness Scale (KSS)^27^ and the Profile of Mood States (POMS)^28^.

In addition to these test bouts, a modified Maintenance of Wakefulness Test (MWT)^29–33^—a physiological measure of the ability to resist sleep—was administered during the SR experiments at baseline and after five nights of SR (a single trial was conducted between 1430h and 1600h) using a standard recording montage. Before each trial, the lights were dimmed to <10 lux and subjects were instructed, “Keep your eyes open and try not to fall asleep.” Each trial was terminated at the first microsleep (10 seconds of theta activity) determined by the C_3_-A_2_ derivation or at 30 minutes if sleep onset did not occur. MWT scores represented either the time (minutes) to microsleep initiation or 30 minutes (if no microsleep occurred).

### Data Analysis

Response to sleep loss was assessed using the average value of data collected every 2h from 2200h/0000h to 1800h/2000h during TSD and using the average value of data collected every 2h from 0800h to 2000h after the fifth night of SR. For the TSD experiments, all subjects (N = 23) were included in the neurobehavioral analyses. For the SR experiments, all subjects (N = 24) were included in the PVT and KSS analyses, n = 23 subjects were included in the MWT analyses, and n = 19 subjects were included in the DSST, DS, and POMS analyses, since these latter measures were not collected in one study. For the sleep loss difference from baseline variables, the last baseline night was used for analyses.

Paired-t-tests (two-tailed, comparing outcome measures for TSD-TSD and SR-SR exposures) and intraclass correlation coefficients^34^ (ICC: two-way mixed, absolute agreement, average measures) assessed the interindividual differences and intraindividual stability of neurobehavioral responses (absolute and sleep loss difference from baseline) to TSD and SR (SPSS v21). The following ranges characterize ICCs and reflect the stability of interindividual differences: 0.0–0.2 (slight); 0.2–0.4 (fair); 0.4–0.6 (moderate); 0.6–0.8 (substantial); and 0.8–1.0 (almost perfect)^34^. Spearman’s rho assessed the relative rank of individuals across measures.

### Data Availability

The datasets analyzed during the current study are available from the corresponding author on reasonable request.

## Results

Sleep duration and timing (assessed using wrist actigraphy) did not differ during the week prior to SR-SR (mean ± SD, Study 1 sleep duration: 8.10h ± 0.67h, Study 2 sleep duration: 8.02h ± 0.49h, P = 0.61; Study 1 sleep onset: 23.61h ± 0.78h, Study 2 sleep onset: 23.65h ± 0.86h, P = 0.84; Study 1 sleep offset: 7.71h ± 0.94h, Study 2 sleep offset, 7.65h ± 0.81h, P = 0.67) or TSD-TSD exposures (mean ± SD, Study 1 sleep duration: 8.18h ± 0.66h, Study 2 sleep duration: 7.97h ± 0.49h, P = 0.07; Study 1 sleep onset: 23.64h ± 0.96h, Study 2 sleep onset: 23.73h ± 0.85h, P = 0.46; Study 1 sleep offset: 7.84h ± 0.86h, Study 2 sleep offset: 7.69h ± 0.78h, P = 0.17). Chronotype^22^ also did not differ prior to SR-SR or TSD-TSD exposures (Ps > 0.05).

### Cognitive Performance

**Figure 1:**
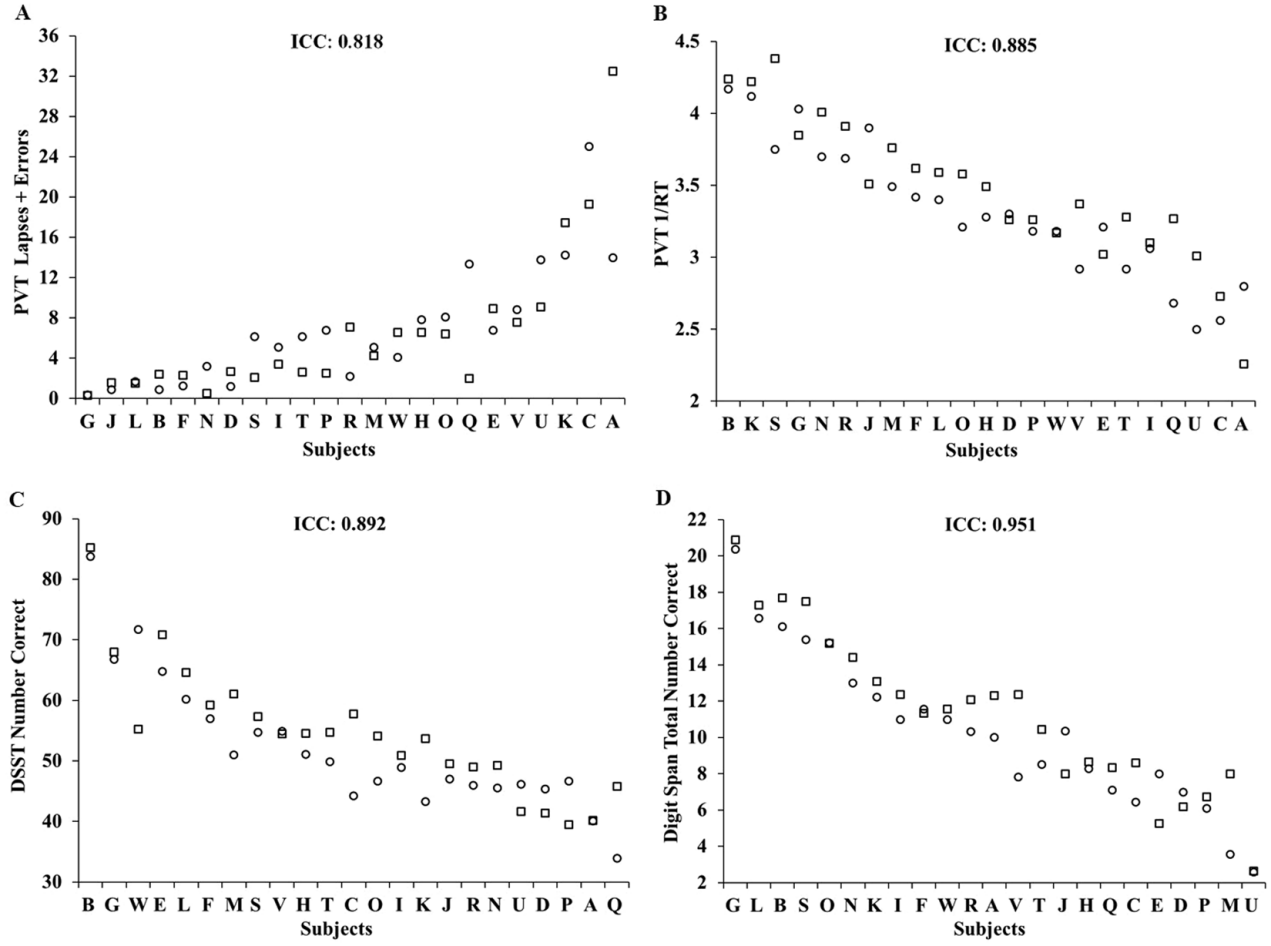
Individual differences and substantial phenotypic stability of cognitive measures to repeated TSD exposures across months to years. Neurobehavioral vulnerability to TSD exposures, separated by 27–2,091 days (mean: 444 days; median: 210 days), showed trait-like stability across performance measures, as evident by almost perfect intraclass correlation coefficients (ICCs): (**A**) 10-minute PVT lapses and errors, ICC = 0.818; (**B**) 10-minute PVT 1/RT, ICC = 0.885; (**C**) DSST number correct, ICC = 0.892; and (**D**) DS total number correct, ICC = 0.951. In all graphs, subjects (denoted individually with letters that correspond to Table 1) are ordered left to right from least to greatest TSD response as determined by the average of the Study 1 (circle) and Study 2 (square) performances. See text for ICC ranges.

Cognitive performance was consistent across the two exposures to TSD, with almost perfect intraclass correlation coefficients (ICCs) for all measures (Fig. 1): 10-minute PVT lapses and errors: 0.818; 10-minute PVT response speed (1/RT): 0.885; DSST: 0.892; and DS: 0.951. TSD performance was consistent within individuals across exposures for PVT lapses and errors (P = 0.780) and DSST (P = 0.077), but showed a difference for PVT 1/RT (P = 0.023) and DS (P = 0.007). There were large phenotypic individual differences in cognitive responses (average of study 1 and study 2 responses) across subjects: average 10-minute PVT lapses and errors ranged from 0.35–23.25; average 10-minute PVT 1/RT ranged from 2.53–4.21 seconds; average DSST performance ranged from 39.58–84.49 correct responses; and average DS performance ranged from 2.61–20.63 correct responses. ICC analyses of the difference of TSD performance from baseline ranged from fair to substantial: PVT lapses and errors: 0.772; PVT 1/RT: 0.300; DSST: 0.566; and DS: 0.484. The change in performance from baseline to TSD was consistent within individuals across exposures for PVT lapses and errors (P = 0.581), PVT 1/RT (P = 0.549), DSST (P = 0.821) and DS (P = 0.768). The average of study 1 and study 2 difference scores from TSD to baseline also revealed large individual differences: PVT lapses and errors ranged from 0.26–20.13; PVT 1/RT ranged from −0.86 to −0.11 seconds; DSST ranged from −21.91–1.10 correct responses; and DS ranged from −4.26–1.88 correct responses.

**Figure 2:**
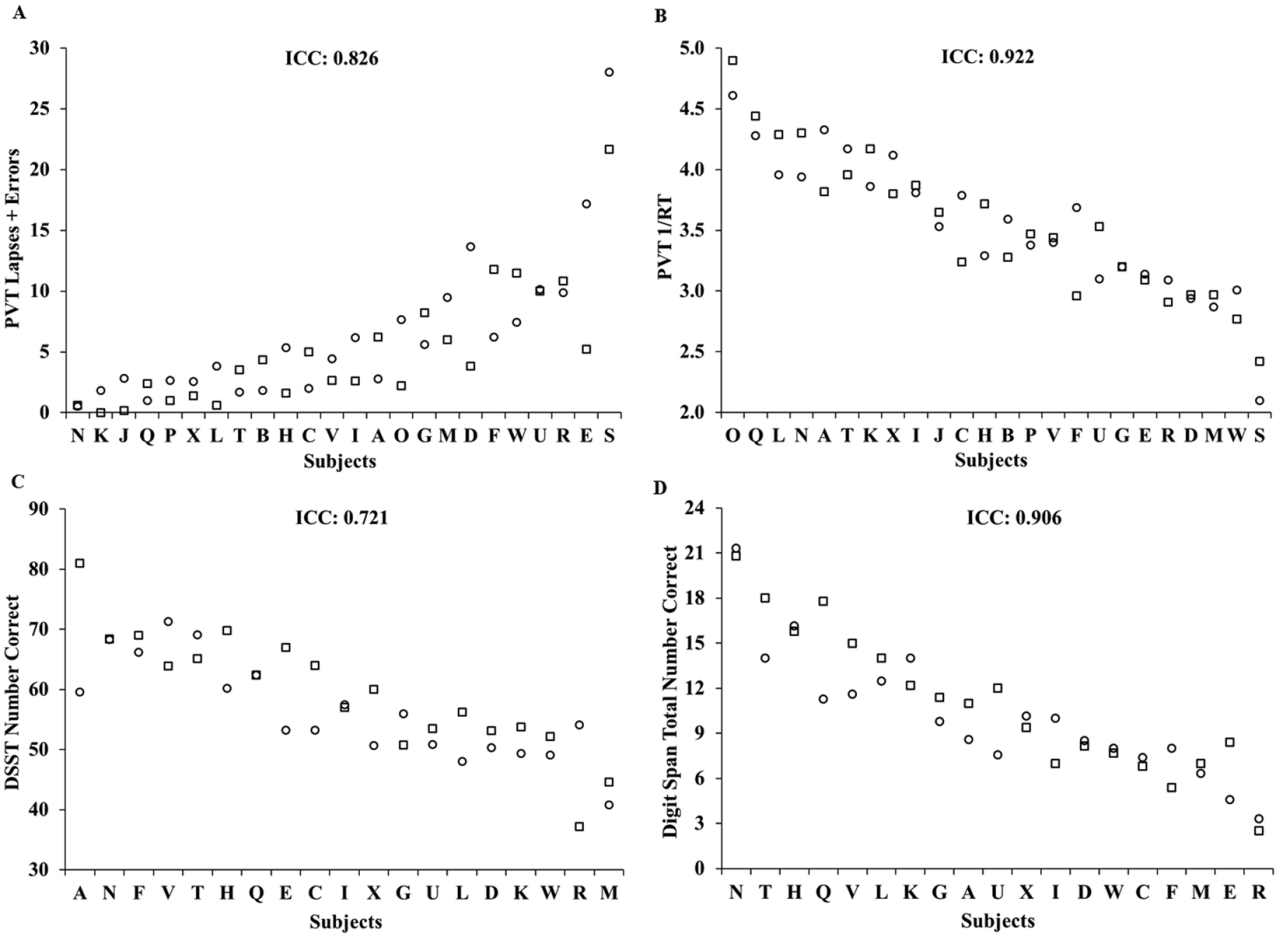
Individual differences and substantial phenotypic stability of cognitive measures to repeated SR exposures across months to years. Neurobehavioral vulnerability to SR exposures, separated by 78–3,058 days (mean: 935 days; median: 741 days), showed trait-like stability across performance measures, as evident by substantial to almost perfect intraclass correlation coefficients (ICCs): (**A**) 10-minute PVT lapses and errors, ICC = 0.826; (**B**) 10-minute PVT 1/RT, ICC = 0.922; (**C**) DSST number correct, ICC = 0.721; and (**D**) DS total number correct, ICC = 0.906. In all graphs, subjects (denoted individually with letters that correspond to Table 2) are ordered left to right from least to greatest SR response as determined by the average of the Study 1(circle) and Study 2 (square) performances. See text for ICC ranges.

Cognitive performance was also consistent across the two exposures to chronic SR, with ICCs ranging from substantial to almost perfect (Fig. 2): PVT lapses and errors: 0.826; PVT 1/RT: 0.922; DSST: 0.721; and DS: 0.906. SR performance was consistent within individuals across exposures for PVT lapses and errors (P = 0.148), PVT 1/RT (P = 0.985), DSST (P = 0.128) and DS (P = 0.146). There were also large phenotypic individual differences in cognitive responses to SR (average of study 1 and study 2 responses) across subjects: average PVT lapses and errors ranged from 0.55–24.84; PVT 1/RT ranged from 2.26–4.46 seconds; average DSST ranged from 42.72–70.30 correct responses; and average DS performance ranged from 2.92–21.07 correct responses. ICC analyses of the difference of SR performance from baseline ranged from slight to substantial: PVT lapses and errors: 0.714; PVT 1/RT: 0.670; DSST: 0.507; and DS: 0.067. The change in performance from baseline to SR was consistent within individuals across exposures for PVT lapses and errors (P = 0.538), PVT 1/RT (P = 0.660), DSST (P = 0.128) and DS (P = 0.476). The average of study 1 and study 2 difference scores from SR to baseline also revealed large individual differences: PVT lapses and errors ranged from −0.09–21.84; PVT 1/RT ranged from −1.14–0.05 seconds; DSST ranged from −15.69–9.15 correct responses; and DS ranged from −3.74–2.52 correct responses.

### Subjective Sleepiness, Fatigue and Vigor

**Figure 3:**
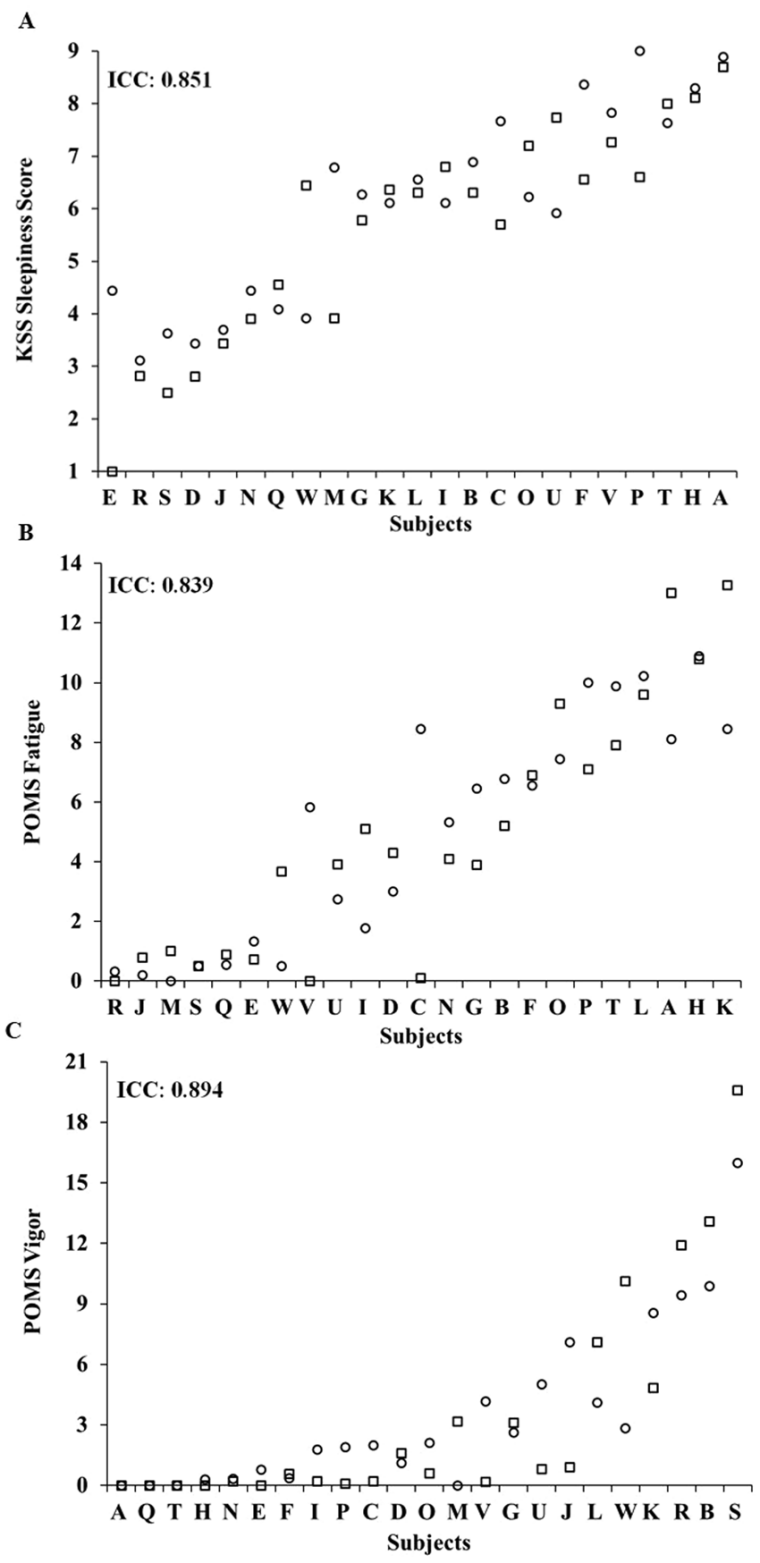
Individual differences and substantial phenotypic stability of subjective measures to repeated TSD exposures across months to years. Neurobehavioral vulnerability to TSD exposures, separated by 27–2,091 days (mean: 444 days; median: 210 days), showed trait-like stability across subjective measures, as evident by almost perfect intraclass correlation coefficients (ICCs): (**A**) KSS score, ICC = 0.851; (**B**) POMS fatigue, ICC = 0.839; and (**C**) POMS vigor, ICC = 0.894. In all graphs, subjects (denoted individually with letters that correspond to Table 1) are ordered left to right from least to greatest TSD response as determined by the average of the Study 1 (circle) and Study 2 (square) ratings. See text for ICC ranges.

Subjective ratings of sleepiness, fatigue and vigor were stable across the two exposures to TSD, with almost perfect ICCs for all subjective measures (Fig. 3): KSS: 0.851; POMS Fatigue (POMS-F): 0.839; and POMS Vigor (POMS-V): 0.894. TSD subjective ratings were consistent within individuals across exposures for KSS (P = 0.130), POMS-F (P = 0.823) and POMS-V (P = 0.885). There were large phenotypic individual differences in subjective responses (average of study 1 and study 2 responses) across subjects: average KSS ratings ranged from 2.72–8.80; average POMS-F ratings ranged from 0.17–10.86; and average POMS-V ratings ranged from 0–17.8. ICC analyses of the difference of TSD subjective ratings from baseline ranged from moderate to almost perfect: KSS: 0.582; POMS-F: 0.842; and POMS-V: 0.718. The change in subjective ratings from baseline to TSD was consistent within individuals across exposures for KSS (P = 0.990), POMS-F (P = 0.951) and POMS-V (P = 0.937). The average of study 1 and study 2 difference scores from TSD to baseline also revealed large individual differences: KSS average differences ranged from 0.18–5.51; POMS-F average differences ranged from −4.34–2.42; and POMS-V average differences ranged from −14.21–0.28.

**Figure 4:**
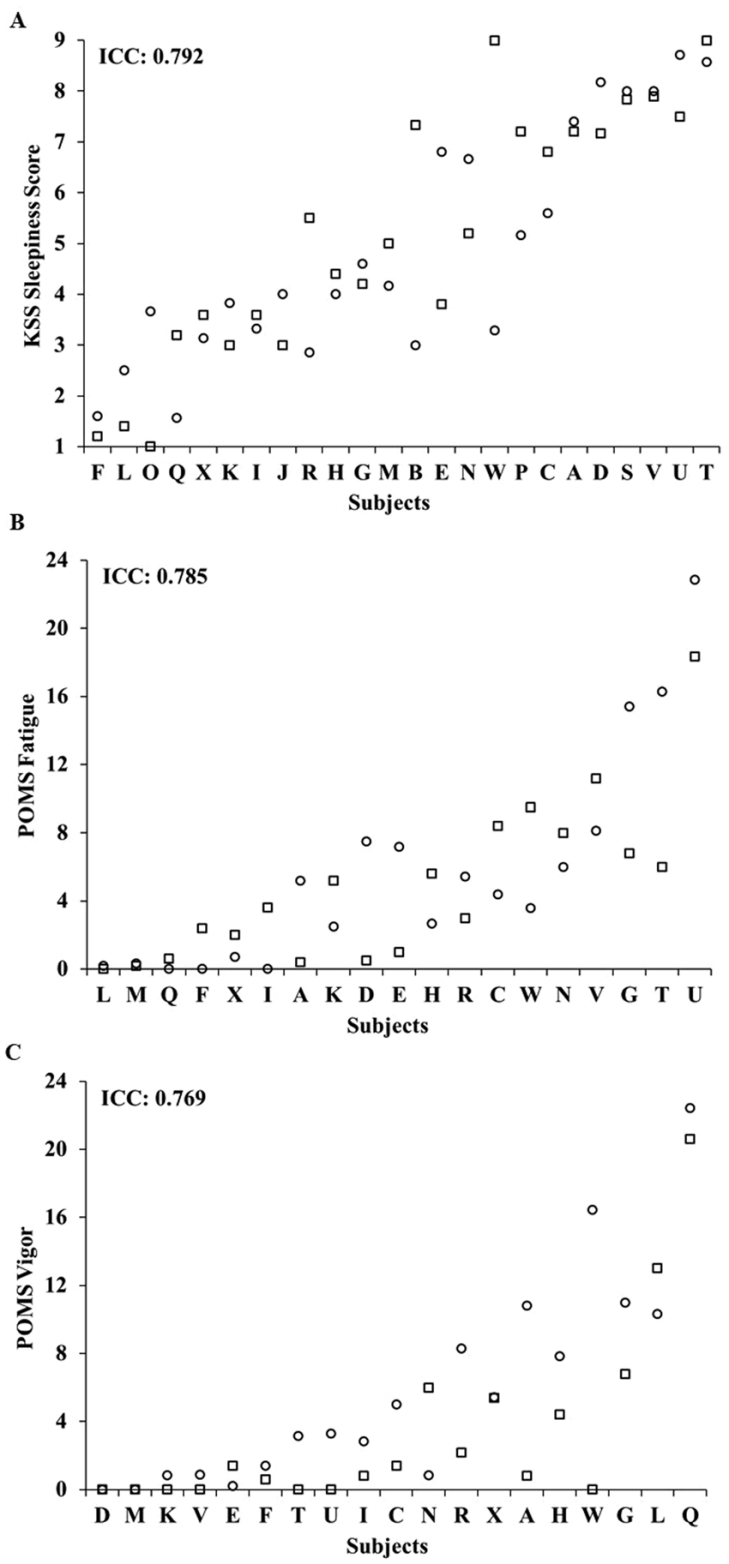
Individual differences and substantial phenotypic stability of subjective measures to repeated SR exposures across months to years. Neurobehavioral vulnerability to SR exposures separated by 78–3,058 days (mean: 935 days; median: 741 days), showed trait-like stability across subjective measures as evident by substantial intraclass correlation coefficients (ICCs): (**A**) KSS score, ICC = 0.792; (**B**) POMS fatigue, ICC = 0.785; and (**C**) POMS vigor, ICC = 0.769. In all graphs, subjects (denoted individually with letters that correspond to Table 2) are ordered left to right from least to greatest SR response as determined by the average of the Study 1 (circle) and Study 2 (square) ratings. See text for ICC ranges.

Subjective ratings of sleepiness, fatigue and vigor were also stable across the two exposures to SR, with substantial ICCs for all measures (Fig. 4): KSS: 0.792; POMS-F: 0.785; and POMS-V: 0.769. SR subjective ratings were consistent within individuals across exposures for KSS (P = 0.517) and POMS-F (P = 0.458) but showed a difference for POMS-V (P = 0.032). There were large phenotypic individual differences in subjective responses (average of study 1 and study 2 responses) across participants: average KSS ratings ranged from 1.40–8.79; average POMS-F ratings ranged from 0.09–20.60; and average POMS-V ratings ranged from 0–21.52. ICC analyses of the difference of SR subjective ratings from baseline ranged from moderate to almost perfect: KSS: 0.682; POMS-F: 0.800; and POMS-V: 0.401. The change in subjective ratings from baseline to SR was consistent within individuals across exposures for KSS (P = 0.480), POMS-F (P = 0.717) and POMS-V (P = 0.785). The average of study 1 and study 2 difference scores from SR to baseline also revealed large individual differences: KSS average differences ranged from −0.39–6.17; POMS-F average differences ranged from 0.09–20.60; and POMS-V average differences ranged from −13.65–3.0.

### Physiologic Alertness

Physiologic alertness was measured during SR using the Maintenance of Wakefulness Test (MWT; MWTs were not performed during the TSD protocols). MWT latency was moderately consistent across the SR exposures (ICC = 0.443) and was consistent within individuals across exposures (P = 0.194). There were large phenotypic individual differences in MWT responses (average of study 1 and study 2 responses) across participants: average latency ranged from 3.72–30 minutes (30 minutes indicates that no microsleeps occurred for the duration of the test). ICC analysis of the difference of MWT latency from baseline was slight (ICC = −0.218), although the change in MWT latency from baseline to SR was consistent within individuals across exposures (P = 0.771). The average of study 1 and study 2 difference scores from SR to baseline also revealed large individual differences: average latency difference ranged from −18.68–3.77 minutes.

### Neurobehavioral Measures: Relative Rank Relationships

Cognitive performance and subjective ratings showed consistency across the different neurobehavioral test responses when averaging exposures for both TSD (Table 3) and SR (Table 4). During TSD (Table 3), PVT lapses and errors was positively correlated with PVT 1/RT (ρ = 0.60, P = 0.003) and DS (ρ = 0.46, P = 0.03). PVT 1/RT was also positively correlated with DS (ρ = 0.57, P = 0.004) and inversely correlated with POMS-V (ρ = −0.61, P = 0.002). DSST was positively correlated with DS (ρ = 0.44, P = 0.04) and KSS was positively correlated with POMS-F (ρ = 0.69, P < 0.001). There were no significant correlations between PVT lapses and errors and DSST, KSS, POMS-V or POMS-F, or between PVT 1/RT and DSST, KSS, POMS-V or POMS-F (all P > 0.05). DSST and DS were not significantly correlated with KSS, POMS-V, or POMS-F, and POMS-V was not significantly correlated with POMS-F or KSS (all P > 0.05).

**Table 3:** Spearman’s Rank Correlation Coefficients for Neurobehavioral Measures for the TSD Exposures.

	PVT Lapses and Errors	PVT 1/RT	DSST	DS	KSS	POMS-V	POMS-F
PVT Lapses and Errors		0.60*	0.34	0.46*	0.29	−0.26	0.08
PVT 1/RT	0.60*		0.38	0.57*	0.39	−0.61*	0.02
DSST	0.34	0.38		0.44*	0.05	−0.38	0.01
DS	0.46*	0.57*	0.44*		0.07	−0.38	−0.28
KSS	0.29	0.39	0.05	0.07		−0.37	0.69*
POMS-V	−0.26	−0.61*	−0.38	−0.38	−0.37		−0.30
POMS-F	0.08	0.02	0.01	−0.28	0.69*	−0.30	

*P < 0.05.

**Table 4:** Spearman’s Rank Correlation Coefficients for Neurobehavioral Measures for the SR Exposures.

	PVT Lapses and Errors	PVT 1/RT	DSST	DS	KSS	POMS-V	POMS-F
PVT Lapses and Errors		0.74*	0.76*	0.31	0.27	−0.25	0.18
PVT 1/RT	0.74*		0.70*	0.54*	0.39	−0.38	0.23
DSST	0.76*	0.70*		0.48*	−0.11	−0.31	−0.23
DS	0.31	0.54*	0.48*		−0.14	−0.11	−0.14
KSS	0.27	0.39	−0.11	−0.14		−0.35	0.73*
POMS-V	−0.25	−0.38	−0.31	−0.11	−0.35		−0.07
POMS-F	0.18	0.23	−0.23	−0.14	0.73*	−0.07	

*P < 0.05.

During SR (Table 4), PVT lapses and errors was positively correlated with PVT 1/RT (ρ = 0.74, P < 0.001) and DSST (ρ = 0.76, P < 0.001). Similarly, PVT 1/RT was positively correlated with DSST (ρ = 0.70, P = 0.001) and DS (ρ = 0.54, P = 0.02); DSST and DS were positively correlated (ρ = 0.48, P = 0.04) and KSS was positively correlated with POMS-F (ρ = 0.73, P < 0.001). There were no significant correlations between PVT lapses and errors and DS, KSS, POMS-V or POMS-F, or between PVT 1/RT and POMS-V, POMS-F or KSS (all P > 0.05). POMS-V was not significantly correlated with POMS-F or KSS (all P > 0.05).

## Discussion

This study provides the first evidence of phenotypic stability of neurobehavioral responses to chronic sleep restriction. It also provides evidence for phenotypic inter-individual differences in neurobehavioral responses to acute total sleep deprivation and to sleep restriction across long time intervals (months to years), even when baseline responses are considered. Cognitive performance outcomes and subjective ratings showed consistency across objective measures, and consistency across subjective measures, but not between objective and subjective domains.

Our findings replicate other results comparing TSD-TSD exposures across short time intervals^1,3,10,11^, with respect to range of performance responses and robust ICCs. Our results also are similar to a study comparing TSD and chronic, severe SR^6^, the latter which is essentially equivalent to TSD^35,36^. One other study examined TSD responses across time intervals of 75 days or more (with a range of 2.5–15 months)^15^, and found similar ICC ranges; however, more direct comparisons cannot be made with that dataset, because the individual subject, mean and/or median duration values between exposures for the N = 12 individuals were not specified, and so it is unclear how many of those subjects were on the lower or higher end of the reported range. Notably, for the first time, we show that SR-SR exposures showed comparable ICCs and thus similar stability to TSD-TSD exposures across all neurobehavioral measures.

The ICCs found in this study are also within the ranges of energy balance responses to TSD-SR exposures and to long duration SR-SR exposures^16,17^, within the ranges for polysomnographic sleep and slow-wave energy responses to TSD-TSD exposures^13,14^, and within the ranges for heart rate, heart rate variability, PERCLOS, blink rate, and EEG alpha power responses to TSD-TSD exposures^15^.

We examined the role of baseline performance and ratings to determine whether these affected ICC values during sleep loss. Inclusion of baseline values resulted in noticeably lower ICC values for PVT 1/RT, DSST and DS in both sleep loss conditions, for KSS in the TSD-TSD condition, and for POMS-V and MWT in the SR-SR condition. By contrast, ICCs for PVT lapses and errors and POMS-F were unaffected for either type of sleep loss condition. Our results suggest the stable individual differences observed for these outcome measures are not due to sleep loss alone, a finding also reported by others^6,10,37^. For example, these ICC reductions may be due to learning effects for some of the tasks, such as the DS, which does not change significantly with sleep loss^29–32^, and which showed baseline differences between TSD-TSD exposures. Reductions in the PVT 1/RT ICC metric may be due to the sensitivity of this variable to sleep loss^25,38^, and due to baseline differences between TSD-TSD exposures that may have been affected by a slight slowing of response speed with increasing age. Importantly, although differences in baseline neurobehavioral functioning contributed to ICCs during sleep loss, they did not negate the systematic individual differences in the magnitude of impairment observed during sleep deprivation.

For TSD-TSD and SR-SR exposures, PVT lapses and errors and PVT 1/RT measures were highly correlated with each other and with DSST and/or DS, and KSS scores were highly correlated with POMS-F scores. Overall, cognitive tests were not correlated strongly with either POMS measure or with the KSS. There were only minor differences between the two types of sleep loss (i.e., TSD and SR) with respect to the relationships of measures with each other. Our findings are consistent with previous research, which found objective assessments of performance did not show congruence with subjective ratings^2,10,11,18,19^, and indicate that subjects’ ranking in terms of vulnerability or resistance varies depending on the task or measure.

Individual differences have not been accounted for by demographic factors, by circadian chronotype or by sleep need; moreover, psychometric scales have not reliably identified neurobehaviorally vulnerable individuals^2,10^. A study using monozygotic and dizygotic twin pairs found substantial differences in PVT responses to TSD—56.2% of the total variance in monozygotic twins was due to variance between pairs compared with 14.5% in dizygotic twins, indicating the response to acute TSD is a highly stable, genetically determined trait^39^. In addition, candidate gene and omic studies highlight a role for various biological factors underlying individual differences to sleep loss^40,41^, although future studies are needed to further explore the biological underpinnings of such phenotypically stable responses.

Although these data were collected in a laboratory setting, these findings have implications for military personnel, health care workers, truck drivers and workers in other applied settings in which sleep loss is common and in which individual differences in vulnerability to the cognitive and fatigue decrements caused by sleep loss could have potentially dangerous consequences^37,42,43^. We show, for the first time, robust differential vulnerability and phenotypic stability of neurobehavioral responses to two commonly experienced types of sleep loss across long time intervals, heralding the use of biomarkers and countermeasures for prediction and mitigation of this critical vulnerability.
